# Inside the Framework: Structural Exploration of Mesoporous Silicas MCM-41, SBA-15, and SBA-16

**DOI:** 10.3390/ma18153597

**Published:** 2025-07-31

**Authors:** Agnieszka Karczmarska, Wiktoria Laskowska, Danuta Stróż, Katarzyna Pawlik

**Affiliations:** 1 Institute of Nuclear Physics Polish Academy of Sciences, PL-31-342 Krakow, Poland; 2 Faculty of Production Engineering and Materials Technology, Częstochowa University of Technology, PL-42-201 Częstochowa, Poland; 3 Institute of Materials Engineering, University of Silesia in Katowice, PL-41-500 Chorzów, Poland

**Keywords:** ordered mesoporous silica materials, SBA-15, MCM-41, SBA-16, nitrogen physisorption, low-angle X-ray diffraction

## Abstract

In the rapidly evolving fields of materials science, catalysis, electronics, drug delivery, and environmental remediation, the development of effective substrates for molecular deposition has become increasingly crucial. Ordered mesoporous silica materials have garnered significant attention due to their unique structural properties and exceptional potential as substrates for molecular immobilization across these diverse applications. This study compares three mesoporous silica powders: MCM-41, SBA-15, and SBA-16. A multi-technique characterization approach was employed, utilizing low- and wide-angle X-ray diffraction (XRD), nitrogen physisorption, and transmission electron microscopy (TEM) to elucidate the structure–property relationships of these materials. XRD analysis confirmed the amorphous nature of silica frameworks and revealed distinct pore symmetries: a two-dimensional hexagonal (*P6mm*) structure for MCM-41 and SBA-15, and three-dimensional cubic (*Im$\bar{3}$m*) structure for SBA-16. Nitrogen sorption measurements demonstrated significant variations in textural properties, with MCM-41 exhibiting uniform cylindrical mesopores and the highest surface area, SBA-15 displaying hierarchical meso- and microporosity confirmed by NLDFT analysis, and SBA-16 showing a complex 3D interconnected cage-like structure with broad pore size distribution. TEM imaging provided direct visualization of particle morphology and internal pore architecture, enabling estimation of lattice parameters and identification of structural gradients within individual particles. The integration of these complementary techniques proved essential for comprehensive material characterization, particularly for MCM-41, where its small particle size (45–75 nm) contributed to apparent structural inconsistencies between XRD and sorption data. This integrated analytical approach provides valuable insights into the fundamental structure–property relationships governing ordered mesoporous silica materials and demonstrates the necessity of combined characterization strategies for accurate structural determination.

## 1. Introduction

In the rapidly evolving fields of materials science, catalysis [1,2], electronics [3,4,5], drug delivery [6,7,8,9], and environmental remediation [10,11,12,13], the development of effective substrates for molecular deposition has become increasingly crucial. These substrates serve as platforms upon which various molecules can be anchored, organized, and activated for specific applications [14,15,16,17]. The interaction between deposited molecules and their supporting substrates fundamentally determines the efficiency, selectivity, and longevity of the resulting systems. The performance of these systems is intrinsically linked to the structural and chemical properties of the substrate material, which directly influence molecular adsorption, diffusion, and reactivity.

Among the various substrate materials available, ordered mesoporous silica (OMS) structures have emerged as particularly promising candidates due to their high surface area, thermal stability, tunable pore sizes, and well-defined pore architectures [18,19,20,21]. The combination of their exceptionally high surface area and the tunability of surface chemistry through functionalization with various organic groups and even large metal-organic molecules makes mesoporous silica an outstanding platform for molecular deposition [22,23,24]. Among the various types of mesoporous silica materials, MCM-41, SBA-15, and SBA-16 are particularly well-known due to their distinct structural features and broad applicability [25,26].

MCM-41 (Mobil Composition of Matter No. 41) exhibits a highly ordered hexagonal arrangement of cylindrical mesopores with high surface area and narrow pore size distribution [27]. These characteristics make it a suitable host for catalysts, adsorbents, and drug delivery systems where precise pore dimensions are critical [28]. In contrast, SBA-15 (Santa Barbara Amorphous type 15) features significantly larger pore sizes and thicker pore walls, which translate into enhanced thermal and mechanical stability [29]. As a result, it is especially suitable for combining with complex molecular systems or nanoparticles in various practical applications [30,31]. Meanwhile, SBA-16 presents a unique three-dimensional cubic architecture characterized by *Im$\bar{3}$m* symmetry, resulting in interconnected spherical cages that facilitate uniform molecular diffusion in all spatial directions [32]. This structure offers distinct advantages for applications requiring enhanced mass transfer efficiency and multidirectional molecular accessibility [33].

A well-established technique for structural characterization is X-ray diffraction (XRD), which is particularly useful for identifying crystalline phases and evaluating long-range order. Key applications of XRD in materials engineering include phase identification, determination of lattice parameters, assessment of crystallinity, estimation of crystallite size, evaluation of residual stress and strain, texture and preferred orientation analysis, as well as monitoring structural changes [34].

X-ray diffraction is effective for characterizing materials with long-range atomic order, but it has limitations when applied to amorphous or nanostructured systems that lack such periodicity [35,36]. These limitations are overcome, among other methods, by the small-angle X-ray scattering (SAXS) technique, which is particularly well-suited for mesoporous materials, as it can detect differences in electron density between the silica framework and the pore spaces [37,38,39]. Despite its effectiveness in characterizing the pore architecture of mesoporous materials, small-angle X-ray scattering has certain limitations. One of the main drawbacks is the need for dedicated instrumentation and software, which are not commonly available in standard laboratory settings. As a result, access to SAXS measurements may be limited by high operational costs and equipment availability. Therefore, it is important to pursue the development of alternative characterization approaches based on conventional XRD techniques. Such methods could potentially offer a more accessible and cost-effective means of obtaining comparable structural information, especially in laboratories without access to advanced SAXS facilities.

In this context, low-angle X-ray diffraction (LAXRD) is particularly important. Here, as in the standard SAXS method, a parallel X-ray beam is used. It is generated using a multilayer parabolic or elliptical mirror, often referred to as a Göbel mirror (GM). In this optical system, X-ray radiation emitted from the tube undergoes multiple reflections within a multilayer structure that has precisely adjusted compositions for the appropriate X-ray wavelength. Due to its design, this mirror works as a Bragg reflector for X-rays. The beam reflects at very small angles from the multilayers and undergoes constructive interference in a defined spatial direction. As a result, the beam becomes both parallel and monochromatic [40]. This beam configuration improves both the precision and the angular resolution of measurements at small scattering angles, as a divergent beam tends to blur the signal, while a parallel beam enables sharper detection of scattering features. Small-angle measurements are typically performed in the angular range of 0.1° to 5°. In addition, the standard divergent beam configuration (Bragg-Brentano) of the X-ray apparatus can be easily and quickly modified to a parallel beam configuration using a GM and precise calibration.

In self-assembled porous structures, long-range pore ordering is frequently observed, where inter-pore distances are constant along specific spatial directions. This resembles long-range atomic ordering in crystals. This ordering is reflected in the LAXRD spectrum, where broad peaks corresponding to the regular arrangement of pores in the material can be identified. Based on the angular positions of these peaks, it is possible to determine the distance between the pores as well as their sizes.

In addition to diffraction analysis, the integration of nitrogen adsorption techniques and transmission electron microscopy (TEM) can provide a more comprehensive understanding of OMS materials [41]. Nitrogen adsorption is a well-established method for probing the textural properties of porous materials. By measuring the adsorption and desorption isotherms of nitrogen gas at cryogenic temperatures, this technique allows the precise determination of key parameters such as specific surface area (using the BET method), total pore volume, and pore size distribution (via BJH or DFT models) [42,43]. TEM, on the other hand, provides high-resolution, direct imaging of the internal mesostructure. It enables the visualization of pore morphology and arrangement at the nanoscale, allowing for a detailed assessment of pore uniformity, wall thickness, and the overall mesostructure [39,44].

In this study, we successfully synthesized three representative types of ordered mesoporous silica (MCM-41, SBA-15, and SBA-16) and subjected them to a comprehensive structural analysis. A central element of our approach was the application of a laboratory-scale X-ray diffraction system equipped with a Göbel mirror, enabling a precise evaluation of the mesostructural order. This was complemented by nitrogen physisorption and transmission electron microscopy, which provided essential information on surface area, pore size distribution, porosity type, and pore wall thickness, as well as direct visualization of internal morphology.

Importantly, this integrative methodology demonstrates that accurate and in-depth characterization of mesoporous silica requires a combination of complementary techniques. Each method probes different structural aspects: XRD characterizes framework ordering, nitrogen sorption assesses textural properties, and TEM provides spatial resolution of internal structures. The synergy of these techniques not only enables a more complete understanding of the pore architecture and defect distribution but also helps to reconcile discrepancies in structural interpretation that may occur when relying on a single technique due to its inherent limitations. This work highlights a cost-effective and broadly applicable strategy for advanced mesostructural analysis, making high-quality material characterization more accessible to laboratories with limited instrumentation resources.

## 2. Materials and Methods

### 2.1. Materials

Cetyltrimethylammonium bromide (CTAB), tetraethyl orthosilicate (TEOS), sodium hydroxide (NaOH), hydrochloric acid (HCl), triblock copolymer poly(ethylene oxide)-block-poly (propylene oxide)-block-poly(ethylene oxide) (Pluronic P123, EO_20_PO_70_EO_20_, M_w_ = 5800), and triblock copolymer poly(ethylene oxide)-poly(propylene oxide)-poly(ethylene oxide) (Pluronic F127, EO_106_PO_70_EO_106_, M_w_ = 12,600) were obtained from Sigma-Aldrich Chemical (St. Louis, MO, USA). All other chemicals were of analytical grade and were used without further purification.

### 2.2. Preparation of Mesoporous Silicas

In this study, three different mesoporous silica materials were synthesized in powder form using straightforward and reproducible methodologies.

MCM-41 was prepared following the procedure proposed by Zhao et al. [45]. Initially, 0.5 g of CTAB was dissolved in 240 mL of deionized water under vigorous stirring, followed by the addition of 1.8 mL of 2 M NaOH (aq). After heating the mixture to 80 °C for 30 min, 2.5 mL of TEOS was added, and the solution was stirred for 2 h. The resulting product was subsequently centrifuged and washed three times with ethanol. To remove the CTAB template, the product was calcined at 550 °C for 5 h.

SBA-15 mesoporous silica powder was synthesized according to the method described by Zu et al. [46]. Initially, 2 g of Pluronic P123 was added to a mixture of 15 g of water and 60 g of 2 M aqueous HCl solution in a round-bottom flask. The solution was magnetically stirred at 35 °C for 1 h. Subsequently, 4.25 g of TEOS was added to the block copolymer solution under continued stirring. After stirring for 5 min, the homogeneous solution was kept at 35 °C for 20 h under static conditions and then aged at 90 °C for 2 days. The solid product was collected by filtration, washed with water, and dried at 140 °C for 4 h. To remove the surfactant, the synthesized white powder was calcined at 550 °C for 5 h.

SBA-16 mesoporous silica was synthesized under acidic conditions following the procedure proposed by Hu et al. [47]. In a typical synthesis, 1.0 g of F127 and 0.12 g of CTAB were dissolved with vigorous stirring in a solution of 130 mL of water and 10 mL of concentrated HCl. Then, 4.0 g of TEOS was added with continued vigorous stirring. After 1 h, the obtained gel was transferred to a Teflon-lined vessel, which was placed in a hydrothermal reactor and subsequently heated in an oven at 150 °C for 24 h. The solid product was filtered, washed, dried at room temperature, and finally calcined at 550 °C for 5 h.

### 2.3. Characterization

**Figure 1:**
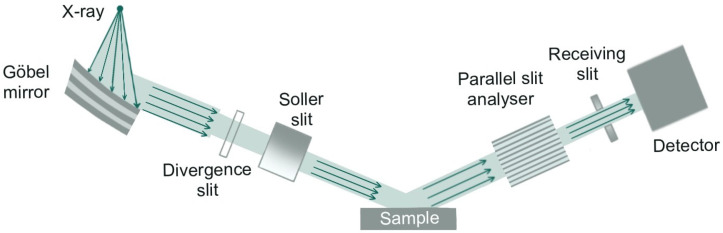
Schematic of the X-ray diffractometer setup, showing the beam path from the source through the Göbel mirror and beam-conditioning optics (based on [40]).

Low-angle X-ray diffraction (LAXRD) patterns were recorded using a Bruker D8 Advance diffractometer (Bruker Corporation, Billerica, MA, USA) operating at 40 kV and 40 mA. The instrument was equipped with a copper X-ray tube (Cu Kα radiation; λ = 1.5418 Å) and a LynxEye silicon strip detector. XRD measurements were carried out using a Göbel mirror to produce a parallel beam. Additionally, a 0.1 mm primary divergence slit and a parallel slit analyzer on the diffracted beam side was applied (Figure 1). The measurements were performed in the 0D mode of the LynxEye detector in a 2Θ range of 0.2° to 5°, with a step size of 0.01° and a counting time of 20 s per step.

The specific surface area was determined by nitrogen adsorption–desorption isotherms measured at 77.4 K using an Autosorb iQ analyzer (Quantachrome Instruments, Boynton Beach, FL, USA). Prior to measurement, samples were degassed at 320 °C for 4 h. The surface area values were calculated using the Brunauer–Emmett–Teller (BET) method, based on adsorption data within the relative pressure range of 0.17–0.23. The microporous volume was determined by the t-plot analysis, and pore size distribution was calculated using the non-local density functional theory (NLDFT) method (cylindrical or cylindrical/sphere pore model using the adsorption branch).

The pore structure was examined using a FEI Tecnai G2 20 X-TWIN transmission electron microscope (TEM) (FEI Company, Hillsboro, OR, USA) operated at 200 kV.

## 3. Results

**Figure 2:**
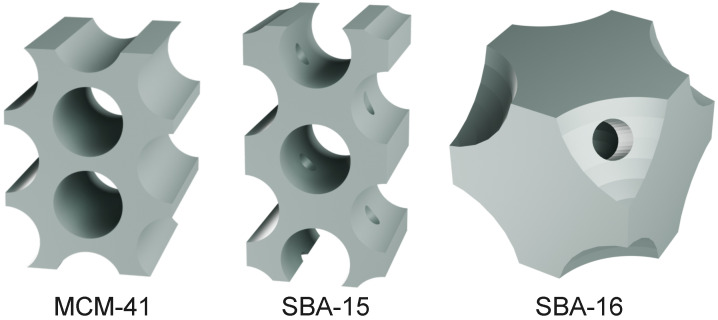
Schematic representation of the pore structures in ordered mesoporous silicas: MCM-41, SBA-15, and SBA-16. Created using Blender 4.4 software [48].

An illustrative representation of the pore architecture of the investigated materials is shown in Figure 2.

MCM-41 exhibits a two-dimensional (2D) hexagonal array of uniform cylindrical mesopores, with pore diameters typically ranging from 2 to 4 nm. Its structure corresponds to the *P6mm* space group and consists of a unidirectional, non-interconnected channel system. SBA-15 also features a 2D hexagonal arrangement (*P6mm*), but with significantly thicker silica walls and larger pore diameters (5–30 nm). Additionally, SBA-15 is characterized by the presence of micropores that interconnect the main mesopore channels, creating a complementary microporous network within the silica walls. In contrast, SBA-16 possesses a three-dimensional (3D) cubic mesostructure, assigned to the *Im$\bar{3}$m* space group, composed of spherical mesopores interconnected through narrow necks. These structural distinctions in unit cell symmetry and pore characteristics are presented in Table 1.

### 3.1. X-Ray Diffraction Analysis

**Figure 3:**
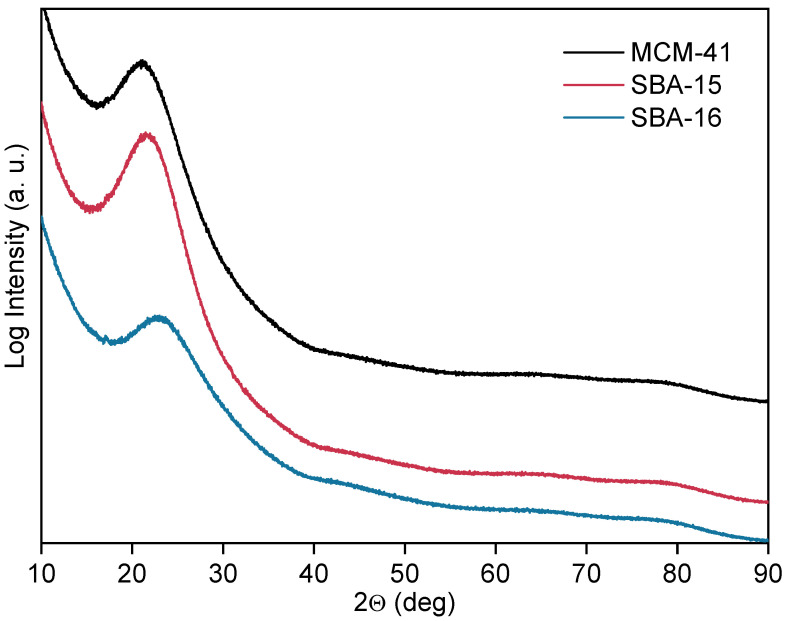
High-angle XRD patterns of MCM-41, SBA-15, and SBA-16.

The X-ray diffractograms recorded in the 2θ range of 10–90° show the broad diffraction peak, characteristic of amorphous silica, that is centered at about 22° (Figure 3) for all samples. No sharp diffraction peaks characteristic of any crystalline component of the sample were present. This confirms that the pore walls are fully amorphous after the synthesis and calcination processes.

**Figure 4:**
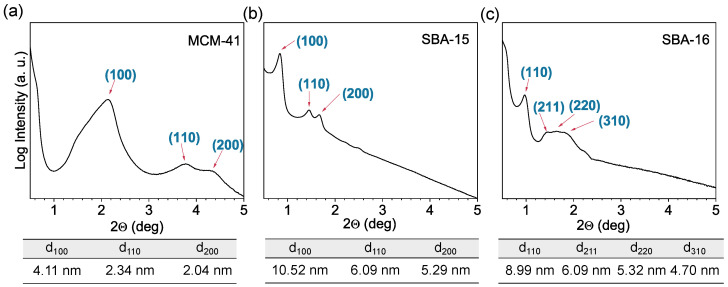
Low-angle X-ray diffraction patterns of mesoporous silica materials: (**a**) MCM-41, (**b**) SBA-15, and (**c**) SBA-16. Miller indices indicate the corresponding diffraction planes, with calculated d-spacing values listed below each diffractogram.

The low-angle XRD patterns for the calcined MCM-41, SBA-15, and SBA-16 samples are presented in Figure 4. The MCM-41 sample exhibits a two-dimensional hexagonal structure (space group *P6mm*) and is usually characterized by a sharp (100) reflection of high intensity and two weaker (110) and (200) reflections [52]. The intensity and sharpness of the low-angle reflections in the XRD patterns reflect the degree of ordering within the mesoporous framework. Sharp and intense peaks signify well-ordered structures, while broader or less intense peaks may indicate partial ordering or structural defects. In the case of our sample, the broad asymmetric (100) peak from ∼1.2° to ∼2.72°, with the maximum at 2.15° and two weak overlapping (110) and (200) reflections (between 3.5° and 4.5°), were recorded (Figure 4a). This significant broadening of the diffraction peaks may be due to the large contribution of defects in the pore structure. This issue will be discussed in more detail later in this paper.

The SBA-15 sample also exhibits a two-dimensional hexagonal mesostructure (space group *P6mm*), characterized by larger pore sizes and thicker pore walls than those of the MCM-41. Its low-angle XRD pattern (Figure 4b) reveals a sharp and intense (100) peak, along with well-resolved (110) and (200) reflections, confirming the high degree of mesostructural ordering [53].

In contrast, the SBA-16 has a three-dimensional cubic structure with *Im$\bar{3}$m* symmetry, which is associated with a cage-like pore network. The XRD pattern of this material displays a distinct (110) reflection, accompanied by three overlapping and broadened peaks corresponding to the (211), (220), and (310) planes (Figure 4c) [32]. Similar to MCM-41, the broadness and shape of these reflections suggest a certain degree of structural inhomogeneity and possibly partial disorder in the SBA-16 framework.

**Figure 5:**
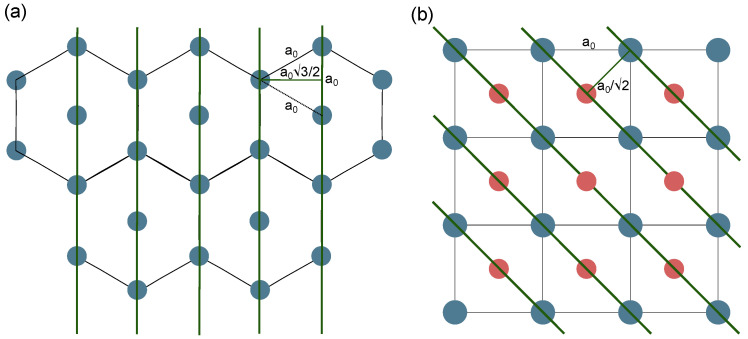
Schematic representation of the *P6mm* (**a**) and *Im$\bar{3}$m* (**b**) structures, with indicated planes for d_100_ and d_110_ calculations, respectively.

The lattice parameters for each mesoporous silica material were determined from the XRD patterns using geometric relationships specific to their respective crystal systems. As illustrated in Figure 5, the hexagonal *P6mm* structure (Figure 5a) and cubic *Im$\bar{3}$m* structure (Figure 5b) exhibit different crystallographic arrangements that require distinct calculation approaches.

For the hexagonal structures, the lattice parameters were calculated using the equation $a_{0}=2d_{100}/\sqrt{3}$, which relates the lattice parameter to the d-spacing of the (100) reflection (Figure 4a,b). This relationship is derived from the hexagonal geometry, where the (100) planes are oriented at specific angles to the unit cell axes, as depicted in Figure 5a. The calculations yielded $a_{0}=4.74$ nm for MCM-41 and $a_{0}=12.15$ nm for SBA-15. The significantly larger lattice parameter for SBA-15 reflects its larger pore diameter and thicker pore walls compared to MCM-41, with both materials maintaining the same hexagonal *P6mm* symmetry.

In contrast, for the cubic structure SBA-16, the lattice parameter $a_{0}=12.71$ nm was calculated using the relationship $a_{0}=\sqrt{2}d_{110}$ (Figure 4c). This equation is specific to the body-centered cubic *Im$\bar{3}$m* symmetry, where the (110) reflection corresponds to the diagonal planes within the cubic unit cell, as shown in Figure 5b. The cubic structure of SBA-16 provides three-dimensional pore connectivity, distinguishing it from the one-dimensional channel systems of the hexagonal materials.

### 3.2. Nitrogen Physisorption Analysis

For further structural analysis, all ordered mesoporous silica materials were analyzed by nitrogen physisorption at 77 K. This analysis provides hysteresis loops whose shapes are closely related to the internal pore architecture of OMS materials [42,54]. Each material exhibits a distinct isotherm shape that reflects its specific pore geometry and interconnectivity, following the International Union of Pure and Applied Chemistry (IUPAC) classification system for physisorption isotherms and hysteresis loops. According to IUPAC recommendations, physisorption isotherms are classified into six main types (Type I–VI), with mesoporous materials typically exhibiting Type IV isotherms. The hysteresis loop classification further subdivides into several categories (H1–H5) based on their shape and the underlying pore structure they represent [55].

**Figure 6:**
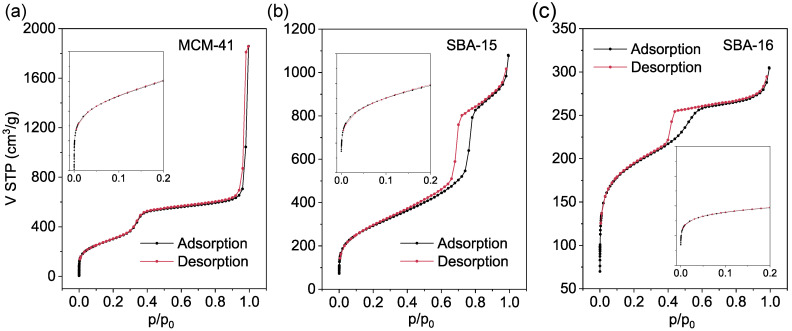
Nitrogen adsorption–desorption isotherms for MCM-41 (**a**), SBA-15 (**b**), and SBA-16 (**c**).

**Figure 7:**
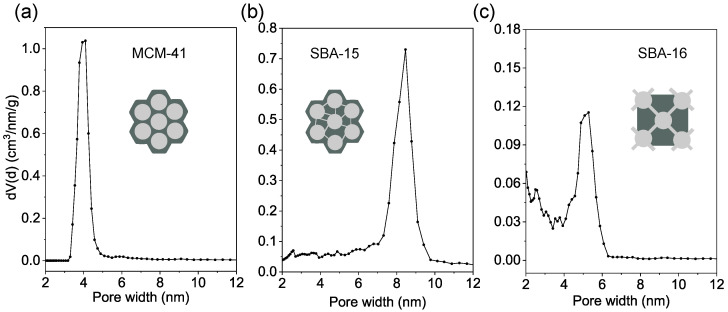
Pore size distributions for MCM-41 (**a**), SBA-15 (**b**), and SBA-16 (**c**) with schematic illustrations of the pore structures (insets).

The three investigated materials exhibit distinctly different isotherm profiles, as shown in Figure 6. MCM-41 (Figure 6a) displays a Type IV(a) isotherm, characteristic of mesoporous materials with uniform, cylindrical, and non-interconnected pores. At low relative pressures (*p*/*p*_0_ < 0.1), the isotherm shows an immediate and steep nitrogen uptake without a clearly defined plateau region, which may suggest the presence of very small mesopores within the material’s structure (see the inset in the figure). This rapid initial adsorption reflects strong adsorbate–adsorbent interactions, typical of materials with a narrow pore size distribution, consistent with the sharp distribution centered around 3.8 nm observed in Figure 7a. The isotherm exhibits a distinct increase in adsorption due to capillary condensation occurring at intermediate relative pressures, and the nearly absent hysteresis loop reflects the highly ordered two-dimensional hexagonal pore arrangement and the independent nature of the pore channels. A gradual increase in nitrogen uptake is also observed near saturation pressure (*p*/*p*_0_ → 1), which may be attributed to interparticle condensation or multilayer adsorption on external surfaces. This effect is commonly reported for MCM-41 and similar materials [56] and does not correspond to the stepwise adsorption mechanism characteristic of Type VI isotherms [55]. Therefore, this isotherm is most appropriately classified as Type IV(a).

SBA-15 (Figure 6b) also displays a Type IV(a) isotherm, but unlike MCM-41, it features a more pronounced H1-type hysteresis loop that indicates the presence of well-defined cylindrical pores with open ends and narrow constrictions [57]. A gradual increase in adsorption is observed in the low-pressure region (*p*/*p*_0_ < 0.1), which may be attributed to the presence of microporous connections between the larger mesopores (see the inset in the figure). This is followed by a sharp capillary condensation step in the intermediate pressure range, with nearly vertical and parallel adsorption/desorption branches, which are typical of uniform mesopore filling. The overall shift of the capillary condensation region toward higher relative pressures compared to MCM-41 reflects the larger pore diameters of SBA-15.

Meanwhile, SBA-16 (Figure 6c) exhibits a Type IV(a) isotherm with a pronounced H2-type hysteresis loop, which is typically associated with materials containing ink-bottle-shaped pores within a complex, interconnected structure [58]. Among the three materials, SBA-16 shows the most gradual nitrogen uptake, with a gently sloping profile extending into the low relative pressure region (*p*/*p*_0_ < 0.1), likely related to multilayer adsorption and possible adsorption in narrow intrawall or interconnecting regions between mesopores (see the inset in the figure). This is followed by a broad capillary condensation step in the intermediate pressure range, characterized by a smoothly increasing adsorption branch and a steep desorption branch, indicative of delayed evaporation due to pore blocking or network effects. The overall shape of the hysteresis loop and the sharp desorption transition are consistent with a three-dimensional mesoporous network composed of larger pore bodies connected by narrower pore necks [55].

The pore size distributions were assessed using the Non-Local Density Functional Theory (NLDFT) method applied to the adsorption branch of the isotherms. The NLDFT approach offers greater accuracy than the classical Barrett–Joyner–Halenda (BJH) method, particularly for mesoporous materials, as it accounts for molecular interactions and intermolecular potentials [43,59,60]. The pore size distributions presented in Figure 7 reveal significant differences between the materials.

MCM-41 shows a narrow pore size distribution (Figure 7a), while SBA-15 exhibits a similarly narrow but shifted distribution toward larger pores (Figure 7b). Notably, SBA-15 also demonstrates the presence of microporosity (at around 2 nm), as illustrated in the schematic inset of Figure 7b, which shows microporous connections between the main cylindrical mesopores. SBA-16 demonstrates a significantly broader pore size distribution (Figure 7c) with dominant pores around 5.1 nm and a smaller fraction near 2.5 nm, reflecting its more complex 3D cubic pore architecture, as illustrated in the inset.

The t-plot method was used to determine the micropore volume by analyzing the microporous region, while the total pore volume was obtained from nitrogen adsorption isotherms at a relative pressure near 1.0. The specific surface area (S_BET_) was evaluated using the Brunauer–Emmett–Teller (BET) method [55]. Wall thickness was calculated as the difference between the lattice parameter $a_{0}$ and the pore width. Textural analysis reveals significant differences in porosity and surface area among the three materials, as shown in Table 2.

**Figure 8:**
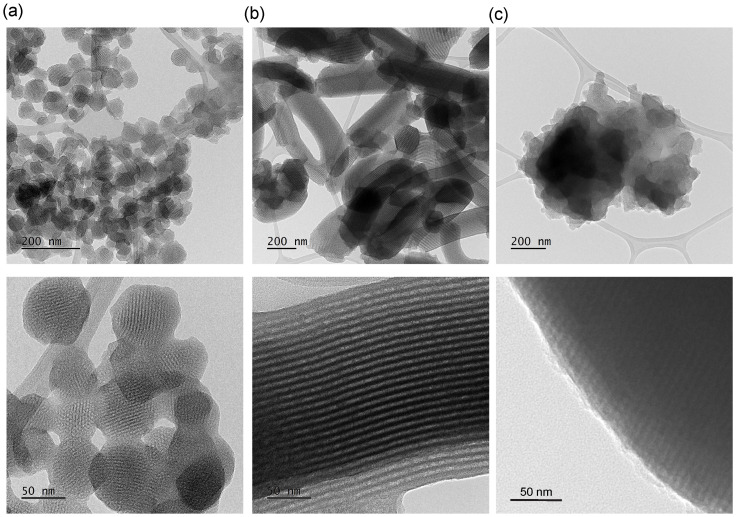
Transmission electron micrographs of MCM-41 (**a**), SBA-15 (**b**), and SBA-16 (**c**).

MCM-41 exhibits the highest specific surface area (1137 m^2^/g) and total pore volume (1.79 cm^3^/g) among the three analyzed materials, with porosity exclusively mesoporous in nature. Although this total pore volume exceeds the typical literature-reported values for MCM-41 (generally < 1.5 cm^3^/g) [61], the discrepancy can be attributed to the presence of additional interparticle porosity. This porosity likely arises from the aggregation of nanoparticles during synthesis, leading to the formation of larger mesopores or even macropores located between the particles. The shape of the hysteresis loop observed in the nitrogen adsorption isotherm (Figure 6a), together with TEM images showing loosely packed spherical particles (Figure 8a), provides strong evidence supporting this interpretation. Consequently, the measured total pore volume reflects contributions from both the intrinsic mesoporous framework and the interparticle voids.

In comparison, SBA-15 displays a moderately high surface area (1056 m^2^/g) and a significantly larger total pore volume (1.67 cm^3^/g), comprising both mesoporous (1.53 cm^3^/g) and microporous (0.14 cm^3^/g) contributions. The presence of microporous volume is consistent with the well-documented intrawall microporosity of SBA-15 reported in the literature [60,62]. Although SBA-15 features thicker pore walls and larger pores than MCM-41, it exhibits a slightly lower surface area due to less dense pore packing.

In contrast, SBA-16 demonstrates the lowest surface area (715 m^2^/g) and total pore volume (0.47 cm^3^/g), reflecting its more complex 3D pore architecture with thicker walls (7.60 nm) that reduce the overall porosity while maintaining structural stability. Notably, although literature reports suggest that SBA-16 may possess a measurable fraction of micropores connecting the mesoporous cages [63], our t-plot analysis of the nitrogen adsorption isotherm returned a micropore volume of zero. This limitation likely arises from the inability of the t-plot method to fully capture the intricate intrawall microporosity inherent in SBA-16’s complex structure. Therefore, employing complementary characterization techniques such as QSDFT modeling or CO_2_ adsorption measurements could provide a more accurate assessment of microporosity and deepen the understanding of the material’s pore architecture [64,65].

Another approach used to estimate the pore diameter of the MCM-41 sample was introduced in [27]. This method relies on a simple geometric relationship between the specific pore volume and pore diameter, assuming an infinite array of cylindrical pores arranged in a hexagonal lattice. In this model, the pore diameter can be calculated from the pore volume V_p_ and the lattice spacing d (obtained from X-ray diffraction measurements), using the following relation: $w=cd{\left(\frac{\rho V_{p}}{1+\rho V_{p}}\right)}^{1/2}$, where $c={\left(\frac{8}{\sqrt{3}\pi }\right)}^{1/2}$ is a constant characteristic of the pore geometry, and ρ = 2.2 g/cm^3^ is the density of pore walls [66]. The pore width of MCM-41, calculated using this method, was 4.43 nm, which is in good agreement with the values obtained using the NLDFT method.

### 3.3. Transmission Electron Microscopy Analysis

All three OMS structures were further visualized using transmission electron microscopy, as presented in Figure 8. The TEM images revealed distinct morphological characteristics of the mesoporous silica materials. MCM-41 particles exhibited a spherical morphology with diameters ranging from 45 to 75 nm, displaying well-ordered hexagonal arrays of parallel cylindrical pores with uniform diameters of approximately 2–4 nm (Figure 8a). SBA-15 samples showed elongated, rod-like particles with lengths of 400–800 nm and widths of 100–250 nm, characterized by highly ordered parallel mesopores arranged in a hexagonal pattern with pore diameters of 6–8 nm (Figure 8b). In contrast, the SBA-16 materials showed irregular particle morphology with sizes ranging from 500 to 800 nm and spherical pores with diameters of 4–6 nm (Figure 8c). All these dimensional parameters were determined using ImageJ 1.54p software based on approximately 50 measurements taken from different areas of each sample to ensure statistical reliability. All three materials exhibited excellent structural regularity, confirming the successful synthesis of highly ordered mesoporous structures. These microscopic observations are in excellent agreement with the results obtained from nitrogen adsorption–desorption isotherms and low-angle X-ray diffraction analyses, which independently confirmed the presence of well-defined mesoporous architectures. The consistency between TEM imaging, BET surface area measurements, and XRD structural characterization provides compelling evidence for the formation of uniform, periodically ordered pore networks with high structural integrity across all three silica materials.

**Figure 9:**
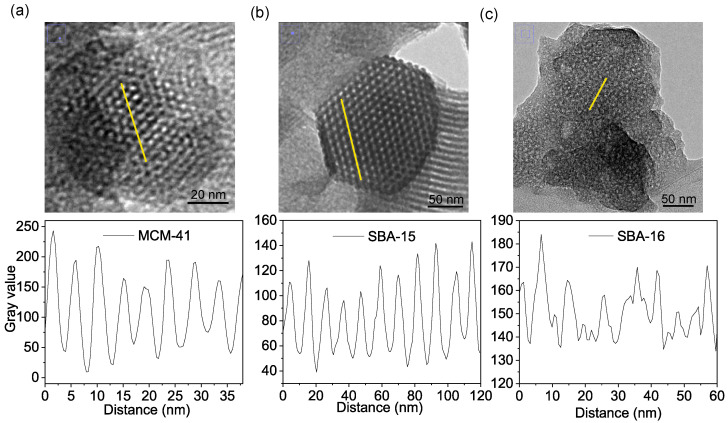
Transmission electron micrographs of MCM-41 (**a**), SBA-15 (**b**), and SBA-16 (**c**), with line profiles through the centers of adjacent pores and corresponding gray-value intensity profiles.

TEM images also served as an alternative method to XRD for determining the lattice parameters of OMS materials, which were previously calculated based on diffraction maxima obtained from XRD experiments (as discussed in Section 3.1). To obtain representative lattice parameter values for each material, we employed the TEM image evaluation approach presented by Beurer et al. [39], which enables the assessment of the maximum number of pores with minimal effort and high reproducibility. Initially, TEM images showing the pore structure in a top-view orientation were selected. Examination of the TEM images reveals grayscale gradations depending on whether a pore wall or an empty pore is observed. Based on these grayscale differences, a line was drawn through the centers of adjacent pores in one direction using ImageJ (Figure 9a). Subsequently, the grayscale intensity was plotted along this distance (Figure 9b). Given the periodicity of the pore structure—where a pore is always followed by a pore wall—the distance between two intensity maxima was interpreted as the lattice parameter. Thus, the positions of the intensity maxima were determined, and the differences between the highest grayscale values were calculated.

For MCM-41, the a_TEM_ lattice parameter was determined to be 4.9 nm, which is approximately 3% larger than the value obtained from XRD data. Similarly, for SBA-15, a value of a_TEM_ = 12.7 nm was obtained, in good agreement with the corresponding diffraction-derived value of 12.2 nm. Acquiring high-quality TEM images with clearly defined pores was more challenging for SBA-16, which is characterized by significantly larger particle dimensions and a more complex 3D cubic structure. The grayscale intensity profile plotted across a selected region revealed that the intensity maxima were less regularly spaced than those in the other materials, likely due to local distortions or imperfect structural ordering. Based on this analysis, the lattice parameter estimated from TEM ranged from approximately 7 nm to 15 nm. Although the XRD-derived value of 12.7 nm falls within this range, the broader variation observed in TEM highlights a notable discrepancy between the two techniques. This divergence can be attributed to the local nature and limited sampling of TEM, which may capture both well-ordered and disordered domains, whereas XRD provides an average value over a much larger volume of material.

Moreover, for the MCM-41 sample, TEM images showed excellent agreement with the results obtained from both X-ray diffraction and nitrogen physisorption analysis. However, it is important to note that data interpretation from these techniques may initially appear inconsistent due to their different analytical sensitivities—XRD reflects the degree of structural ordering in the material, while nitrogen sorption provides information about porosity across the entire volume of the sample. The XRD patterns exhibited broad and asymmetric peaks, which may indicate the presence of defects in the mesoporous framework—particularly at the particle edges, where the structural ordering deteriorates. In contrast, nitrogen adsorption–desorption isotherms revealed a relatively narrow pore size distribution, suggesting a high degree of structural uniformity in the entire sample volume. This apparent discrepancy was clarified through a detailed analysis of TEM images (Figure 8a and Figure 9a). In the case of the MCM-41 sample, the particles are relatively small, with sizes ranging from 45 to 75 nm. As a result, the disordered peripheral regions represent a much larger proportion of each particle. These disordered areas contribute more significantly to the overall signal compared to materials with larger particles, such as SBA-15 or SBA-16, where the particle sizes reach several hundred nanometers. Therefore, only a combined analytical approach involving XRD, nitrogen sorption, and TEM allows for a comprehensive and reliable interpretation of the microstructure of ordered mesoporous silica materials.

## 4. Conclusions

This study presents a comprehensive comparative analysis of three ordered mesoporous silica materials, namely MCM-41, SBA-15, and SBA-16, focusing on their structural, textural, and morphological properties. The use of complementary characterization techniques, including low- and wide-angle XRD, nitrogen physisorption, and transmission electron microscopy (TEM), allowed for a detailed assessment of the relationship between pore architecture, material symmetry, and functional properties. XRD analysis confirmed the amorphous nature of the silica walls in all samples and clearly distinguished the pore symmetries: two-dimensional hexagonal (*P6mm*) for MCM-41 and SBA-15, and three-dimensional cubic (*Im$\bar{3}$m*) for SBA-16.

Nitrogen sorption data revealed significant differences in pore size distributions, surface areas, and porosity types among the three materials, reflecting their underlying structural organization. MCM-41 exhibited a narrow distribution of uniform cylindrical mesopores with the highest surface area and total pore volume. SBA-15 showed both meso- and microporosity, resulting from the presence of interconnecting micropores between larger mesopores, as confirmed by NLDFT analysis. SBA-16 demonstrated the most complex pore structure, with broad pore size distribution and lower overall porosity, attributable to its 3D interconnected cage-like network.

TEM imaging provided detailed insight into the morphology and pore architecture of the synthesized mesoporous silica materials. The technique enabled direct visualization of particle shape, size, and internal ordering, revealing highly organized pore arrangements specific to each material type. Additionally, TEM analysis allowed for the estimation of lattice parameters and highlighted structural gradients within individual particles, particularly in MCM-41 and SBA-16.

The integration of X-ray diffraction (XRD), nitrogen sorption analysis, and TEM proved essential for the reliable characterization of mesoporous materials. Each technique probes different aspects of the material: XRD provides information on framework ordering, nitrogen sorption assesses porosity, and TEM provides direct, spatially resolved structural information. The combined application of these complementary methods provides a more holistic understanding of the mesostructure and helps resolve potential discrepancies in data interpretation that can result from the use of a single analytical technique. This multi-technique approach was particularly valuable for MCM-41, where the small particle size (45–75 nm) resulted in a significant contribution of disordered boundary regions to the overall structural characterization, reconciling the apparent contradiction between broad XRD peaks and narrow pore size distributions observed in sorption measurements. A full understanding of the structural properties of ordered mesoporous silica materials is achievable only through the application of this comprehensive analytical approach.

## Figures and Tables

**Table 1:** Crystallographic properties and pore structure of ordered mesoporous silica materials.

Sample	Space Group	Pore			Interconnectivity	Reference
		Shape	Arrangement	Size (nm)		
MCM-41	*P6mm*	cylindrical	2D hexagonal	2–6.5	–	[49]
SBA-15	*P6mm*	cylindrical	2D hexagonal	5–30	1D channels	[50]
SBA-16	*Im$\bar{3}$m*	spherical	3D cubic	4–9	3D interconnected	[51]

**Table 2:** Textural properties of MCM-41, SBA-15, and SBA-16 determined from N_2_ adsorption measurements.

Sample	S_BET_ (m^2^/g)	Pore Volume (cm^3^/g)			Pore Width (nm)	Wall Thickness (nm)
		Total Volume	Micropore Volume	Mesopore Volume		
MCM-41	1137	1.79	-	1.79	4.0	0.85
SBA-15	1056	1.67	0.14	1.53	8.5	3.78
SBA-16	715	0.47	-	0.47	5.1	7.60

## Data Availability

The datasets used and analyzed during the current study are available from the corresponding author on reasonable request.

## Abbreviations

The following abbreviations are used in this manuscript:

BET	Brunauer–Emmett–Teller method (equation)
BJH	Barret–Joyner–Halenda method
CTAB	Cetrimonium bromide
EDS	Energy dispersive spectroscopy
F127	Pluronic F127
GM	Göbel Mirror
HCl	Hydrochloric acid
IUPAC	International Union of Pure and Applied Chamistry
LAXRD	Low-Angle X-ray Diffraction
MCM-41	Mobil Composition of Matter No. 41
M_w_	Molecular weight
NaOH	Sodium hydroxide
NLDFT	Non-Local Density Functional Theory method
OMS	Ordered Mesoporous Silica
P123	Pluronic P123
SAXS	Small-Angle X-ray Scattering
SBA-15	Santa Barbara Amorphous No. 15
SBA-16	Santa Barbara Amorphous No. 16
S_BET_	BET specific surface area
STP	Standard temperature and pressure
TEM	Transmission electron microscope
TEOS	Tetraethyl orthosilicate
XRD	X-ray diffraction
